# Agricultural waste-based modified biochars differentially affected the soil properties, growth, and nutrient accumulation by maize (*Zea mays* L.) plants

**DOI:** 10.1186/s12870-024-05202-5

**Published:** 2024-06-04

**Authors:** Muhammad Farooq Qayyum, Dur-e-Sameen Khan, Suliman Mohammed Suliman Alghanem, Haifa Abdulaziz Sakit Alhaithloul, Ibtisam Mohammed Alsudays, Muhammad Rizwan, Jean Wan Hong Yong

**Affiliations:** 1 Department of Soil Science, Faculty of Agricultural Sciences & Technology, Bahauddin Zakariya University, Multan, Pakistan; 2 Department of Biology, College of Science, Qassim University, Burydah, 52571 Saudi Arabia; 3 Biology Department, College of Science, Jouf University, Sakaka, Aljouf, 2014 Saudi Arabia; 4 Department of Environmental Sciences, Government College University Faisalabad, Faisalabad, 38000 Pakistan; 5 Department of Biosystems and Technology, Swedish University of Agricultural Sciences, Alnarp, 23456 Sweden

**Keywords:** Pyrolysis, Protonated biochars, Maize crop, Germination, Alkaline soil properties

## Abstract

Biochar (BC) is an organic compound formed by the pyrolysis of organic wastes. Application of BCs as soil amendments has many benefits including carbon sequestration, enhanced soil fertility and sustainable agriculture production. In the present study, we acidified the different BCs prepared from rice straw, rice husk, wheat straw, cotton stalk, poultry manure, sugarcane press mud and vegetable waste; following which, we applied them in a series of pot experiments. Comparisons were made between acidified and non- acidified BCs for their effects on seed germination, soil properties (EC, pH) nutrient contents (P, K, Na) and organic matter. The treatments comprised of a control, and all above-described BCs (acidified as well as non-acidified) applied to soil at the rate of 1% (w/w). The maize crop was selected as a test crop. The results showed that acidified poultry manure BC significantly improved germination percentage, shoot length, and biomass of maize seedlings as compared to other BCs and their respective control plants. However, acidified BCs caused a significant decrease in nutrient contents (P, K, Na) of soil,maize seedlings, and the soil organic matter contents as compared to non- acidified BCs. But when compared with control treatments, all BCs treatments (acidified and non-acidified) delivered higher levels of nutrients and organic matter contents. It was concluded that none of the BCs (acidified and non-acidified) had caused negative effect on soil conditions and growth of maize. In addition, the acidification of BC prior to its application to alkaline soils might had altered soil chemistry and delivered better maize growth. Moving forward, more research is needed to understand the long-term effects of modified BCs on nutrient dynamics in different soils. In addition, the possible effects of BC application timings, application rates, particle size, and crop species have to be evaluated systemtically.

## Introduction

Chemical fertilizers are used conventionally as a viable source of plant nutrients to ensure a sufficient food supply worldwide. However, the continual application of such inorganic fertilizers to soil may affect soil fertility, becoming harmful to the environment in some scenarios, and affecting the natural microbial population [1, 2]. To ensure global food security and to develop sustainable agriculture that is reliable [3, 4] the use of biochar (BC) alone or in combination with chemical fertilizers offers a plausible solution to deliver essential nutrients and biostimulants while maintaining crop production [5–9].

Biochar is a carbon-rich material formed through the pyrolysis of organic wastes [10, 11]. Being more stable and resistant to microbial decomposition, BC can remain stable in soils for thousands of years [12–14] and provide beneficial effects [15] such as the improvement of soil organic matter (SOM) and soil structure [7, 16]. The application of BC in soil may help to increase growth and resilience of plants by improving soil physicochemical properties [6, 17]. It is porous in nature, and it has potential to improve soil bulk density, porosity, moisture contents, water holding capacity and infiltration rate [18–20]. It is well known for improving soil fertility by retaining plant essential nutrients and moisture contents [1,7]. In addition, BC decreases CO_2_ and N_2_O emission [21]; and increases cation exchange capacity (CEC), organic carbon contents and pH of soil [22].

BC can be prepared from wide range of organic feedstocks including crop residues wood-based material, food waste, municipal wastes, sewage sludges and animal wastes and manures [23–26]. In Pakistan, several tons of municipal waste are produced every day which is a source of decomposable and highly nutritious organic materials [27]. Similarly, agricultural-based large amounts of crop residues are left over after harvesting which are burnt in open air and result in release of greenhouse gases into the atmosphere causing adverse environmental impacts [28]. These organic wastes can successfully be converted into BC through pyrolysis.

Since the potential effects of BC depend on the feedstock for BC preparation, pyrolysis conditions, and soil type, its modification can enhance its performance and suitability for specific soils and crops [29, 30, 16]. For example, particle size modification of BC can increase its surface area, porosity, and functional groups, which could also improve its adsorption capacity for nutrients, water, and pollutants [31]. Similarly, the modification with acid can alter the pH, electrical conductivity, and cation exchange capacity of BC, which can affect the properties of alkaline-calcareous soils [32–34]. The acid-modifications can be achieved through washing of BC with strong acids such as hydrochloric acid, phosphoric acid and sulfuric acid which may increase its surface acidity and change the porous structure [32, 34, 25]. Acid treatment also reduces inorganic and metal components of BC. Previous studies reported that acidic BC can increase soil organic matter and soil structure, thereby increasing the water holding capacity and aggregate stability [36]. Moreover, the acidic BC can enhance nutrient dynamics in salt-affected soils by releasing organic acids, chelating metals and or complexing phosphorus compounds [33]. Ramzani et al. reported that S-induced acidification of BC and compost enhanced growth of quinoa in an alkaline soil through modification of soil pH and nutrients availability [37].

Most of the research studies focusing positive effects of BC have been reported for acidic soils. However, very little literature is found related to alkaline soils, especially in Pakistan where more than 40% cultivable area are alkaline, salt-affected and facing serious challenges for crop production [38]. Therefore, there is need to pay attention on the production of BC from various feedstocks and their potential effects on alkaline soils characteristics and plant growth. Since seed germination is a crucial stage in the plant growth, a comparison of acidic and simple BC_s_ for their influence on seed germination, and soil properties would provide valuable insights and plausible solutions for sustainable soil management and crop production in alkaline-calcareous soils.

This study was intended to investigate the potential of simple and acidified BC as a soil amendment in an alkaline calcareous soil with hypotheses, “BC type and modification will influence seed germination, and soil properties”. The key objectives included (1) Preparation and physicochemical characterization of simple and acidified BC prepared from a variety of organic feedstocks; (2) evaluation of the effects of simple and acidified BCs on the germination parameters, biomass, and nutrient uptake of maize seedlings in an alkaline-calcareous soil and (3) evaluation of the effects of simple and acidified BC application on soil pH, electrical conductivity (EC), organic matter, and plant available nutrients.

## Materials and methods

### Experimental location, soil sampling and analyses

Soil sampling was done in the research area of Faculty of Agriculture Sciences and Technology, Bahauddin Zakariya University Multan, Pakistan (Latitude: 030°15ʹ36ʹʹN; Longitude: 071°30ʹ53ʹʹE. These samples were sieved through 2 mm sieves, dried in air overnight and stored in plastic bags. Soil samples were analyzed before and after the experiment for physicochemical properties including EC, pH, nutrient contents such as phosphorus (P), potassium (K) and organic matter contents using standard methods. The soil used in the experiment is an Aridisol, well-drained, weakly structured, and moderate to strongly calcareous in nature. Some of the selected properties of the soil are pH 7.5, EC 0.15 dSm^− 1^, texture silt loam, CaCO_3_ 10%, and organic matter 0.5%.

### Biochar production, acidification, and characterization

Seven organic materials, such as rice straw, rice husk, wheat straw, cotton stalks, poultry manure, sugarcane press mud and vegetable wastes were collected and air dried for 2–3 days. The dried feedstock underwent pyrolysis at a temperature of 350 °C to 400 °C in a vertical silo type reactor. The detailed methodology of BC preparation using this furnace is given in [24]. The heat was provided from outside through natural gas source. After the completion of pyrolysis, the BCs were allowed to cool overnight and ground to pass 5 mm sieve.

All BCs were analyzed for EC, pH, volatile matter, ash contents and nutrient contents such as N, P etc. The pH and EC (µs/m) were determined in slurry of 1:20 (w/v BC and water) using pH and EC meters, respectively. The volatile matter and ash content BC were determined using standard procedure (ASTM D-1762 method with slight modifications of temperature) [39]. Briefly, 1 g of each BC was taken in porcelain crucibles and put in muffle furnace. For volatile matter, the samples were combusted at 450 °C. For ash content, the samples were heated at 550^◦^C respectively. Volatile matter was calculated by estimating the weight loss between 450 °C and 550 °C, and ash content was determined by using the formula:

$$Ash{\text{ }}content{\text{ }}\left( \% \right){\text{ }} = {\text{ }}\left( \begin{gathered} weight{\text{ }}of{\text{ }}sample{\text{ }}left{\text{ }} \hfill \\ after{\text{ }}heating{\text{ }}at{\text{ }}550{\text{ }}^\circ C/ \hfill \\ initial{\text{ }}weight{\text{ }}of{\text{ }}BC{\text{ }}sample \hfill \\ \end{gathered} \right){\text{ }}*100$$

For the determination of nutrient contents such as nitrogen and total phosphorus, BC_s_ were digested in sulfuric acid (H_2_SO_4_) on block digester. The concentrations of N, P and K in digestates were determined using Kjeldahl distillation, spectrophotometer, and flame photometer, respectively. The specific surface area of the BCs was determined using gas (N_2_) adsorption isotherms on NOVA e2200; a Brunauer–Emmett–Teller (BET) based automated multipoint surface analyzer (Quantochrome Instruments, Boynton Beach, FL).

The analyses of BCs show high variability between used BCs for composition. The N content varied from 1.2% (WSB) to 1.9% (PMB), while 1.7% in RSB, CSB, SPB, and VWB. The P content was highest in VWB (0.4%), followed by RSB, RHB, and CSB (0.1%), while lowest in SPB (0.07%). The SPB has the highest volatile matter (91.2%), followed by CSB (78.9%) and WSB (75.5%). PMB has the lowest volatile matter (19.7%). The ash content was highest in PMB (78.4%), followed by RHB (36.8%) and RSB (27.7%). The SPB has the lowest ash content (8.1%). Regarding electrical conductivity, the highest EC value was found in CSB (929 µs/m), followed by PMB (908 µs/m) and SPB (758 µs/m). The lowest values of EC were found in RHB (233 µs/m). The pH values were in VWB (10.7), followed by RSB (9.6) and SPB (9.49), and lowest in CSB (7.1). The specific surface area as measured through BET equation shows that CSB has the highest values (97.0 m^2^/g), followed by RSB (95.0 m^2^/g) and WSB (91.0 m^2^/g), while lowest in VWB (55.0 m^2^/g).

**Table 1:** Physicochemical characteristics of biochars

Biochars	Nitrogen (%)	Phosphorus (%)	Volatile matter (%)	Ash content (%)	Electrical Conductivity (µs/m)	pH	BET Surface Area (m^2^/g)
Rice Straw Biochar	1.7	0.1	71	27.7	368	9.6	95.0
Rice Husk Biochar	1.3	0.1	56	36.8	233	8.15	75.0
Wheat Straw Biochar	1.2	0.08	75.5	23.7	536	8.5	91.0
Cotton Stalk Biochar	1.7	0.1	78.9	10.1	929	7.10	97.0
Poultry Manure Biochar	1.9	0.09	19.7	78.4	908	9.10	65.0
Sugarcane Press-mud Biochar	1.7	0.07	91.2	8.1	758	9.49	72.0
Vegetable Waste Biochar	1.6	0.4	73.3	22.8	648	10.70	55.0

For the acidification of BCs, 30 mL of 1 N HCl solution was added for one replicate of BC treatment (30 g BC). In non-acidified BCs, 30 mL distilled water was added. All mixtures of either BCs-acid or BC-water were shaken on mechanical shaker for 30 min and air dried overnight.

### Description of the experiment

A germination trial was conducted using maize seeds to evaluate and compare both positive and negative effects of acidified and non-acidified BCs on seed germination. Seeds of maize were collected from agriculture extension department of Multan, Punjab Pakistan. The experiment was conducted on alkaline-calcareous soil. Plastic trays were filled with 3.0 kg soil and BC treatments comprising rice straw biochar (RSB), rice husk biochar (RHB), wheat straw biochar (WSB), cotton stalk biochar (CSB), poultry manure biochar (PMB), sugarcane press mud biochar (SPB) and vegetable waste biochar (VWB) were applied to soil at the rate of 1% (30 g) per replicate. The soil without biochar was considered as control. In 28 trays, acidified BCs were added and in the remaining 28 trays non-acidified BCs were added. The 30 mL of acid solution (1 N HCl) was added in four replicates of acidified control treatment. Maize seeds were sown at the rate of thirty (30) seeds per tray. The germination trays were placed in the laboratory (temperature 25 C, light duration 15 h using LED bulbs, humidity 45–50%). The moisture content was maintained up to 60% of the water holding capacity by regular weighing of the trays. Germinated seedlings were counted daily during the germination trial. Harvesting was done after 15 days of germination (total 25 days). After harvesting, three germination parameters including germination percentage (GP), mean emergence time (MET) by using Ellis and Robert [40] equation and coefficient of uniformity of emergence by using Bewely and Black [41] equation were determined.

### Soil and plant analyses

After harvesting plant samples, the soil samples taken from germination pots were dried, passed through a 2 mm sieve, and analyzed for various characteristics. The plant samples were separated into shoot and root, their lengths were measured, and weighed to determine the fresh biomass, and oven dried at 65 °C for the dry biomass. For soil pH and EC, saturated paste of soil and distilled water were prepared. The values of pH_s_ were taken using the pH meter (BANTE PHS 25CW, China). Extracts were taken from the same saturated pastes and readings of EC were taken using EC meter (BANTE DDS 11AW). Soil organic matter content were determined following Walky and Black [42]. Plant available P (Olsen’s P; suitable for alkaline and calcareous soils [43]) and K (ammonium acetate extractable [44]) were determined using spectrophotometer and flame photometer, respectively. The dried plant samples were digested in di-acid mixture (nitric acid: perchloric acid in 2:1 ratio). The digestates were used to determine K and Na concentration using flame photometer, while P concentrations were determined using spectrophotometer [45].

### Statistical analysis

The data for germination, growth, and soil properties were analyzed two-way analysis of variance (ANOVA) and for pairwise comparisons, Tukey’s HSD test was applied. Anova and Tukey test were performed using Statistix 8.1 (Analytical Software). The Pearson correlation was calculated using R.

## Results

### Germination parameters

**Fig. 1:**
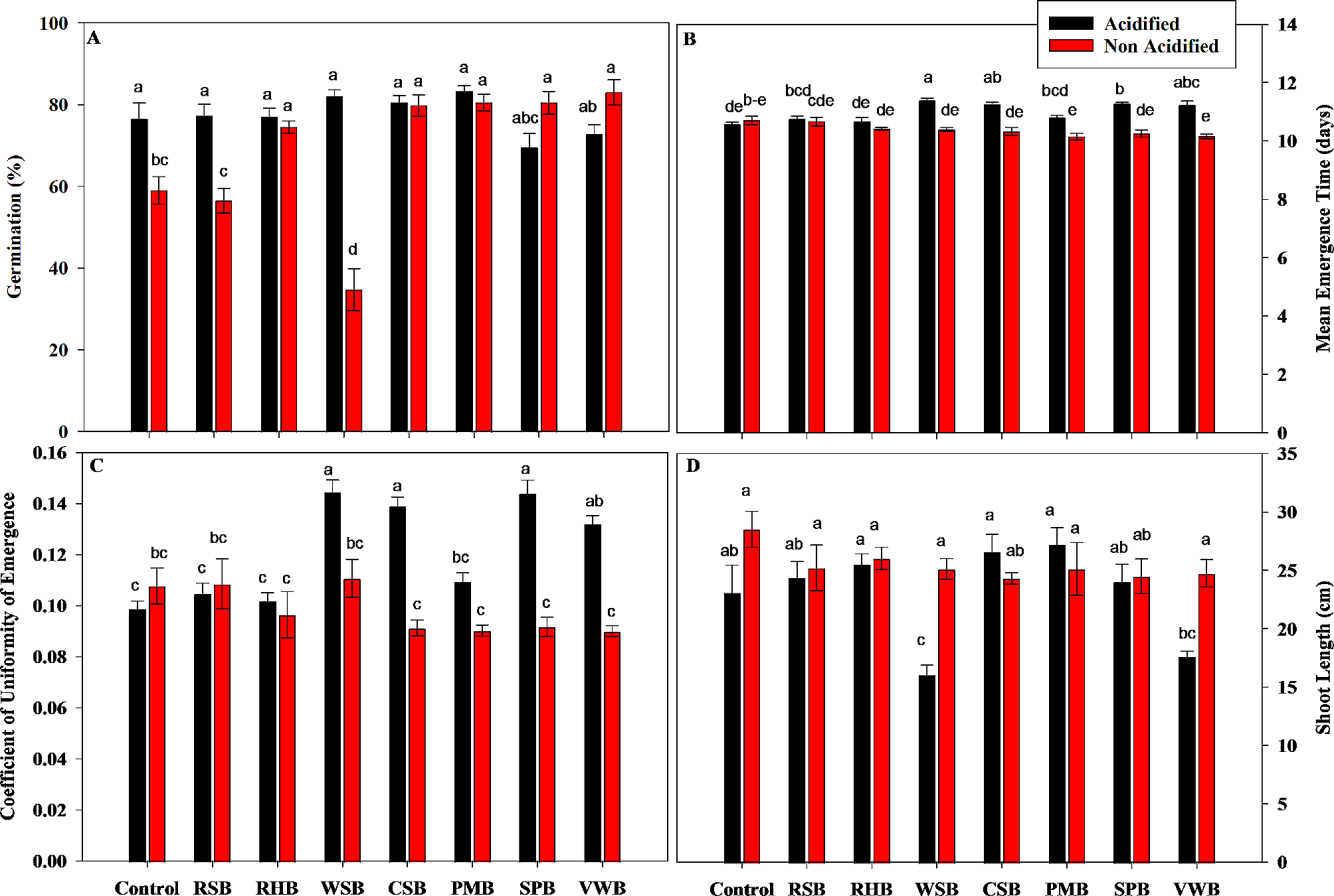
Interactive effects of acidification and BCs (control, rice straw biochar (RSB), rice husk biochar (RHB), wheat straw biochar (WSB), cotton stalk biochar (CSB), poultry manure biochar (PMB), sugarcane press mud biochar and vegetable waste biochar) on germination percentage **(A)**, Mean Emergence Time **(B)**, Coefficient of Uniformity of Emergence **(C)**, and Shoot Length **(D)** of maize. Different letters on bars show significant differences among treatments at both levels of acidification (acidified and non-acidified)

The germination percentage in various treatments ranged from 34 to 83%. The statistical analysis showed that all acidified treatments except SPB and VWB caused significant increase in germination of maize seedlings compared to non-acidified treatments. However, differences among various BC treatments were not significant as compared to control (Fig. 1a).

The statistical analysis showed that the effects of acidified BC treatments on mean emergence time (MET) of maize seedlings were significant as compared to control and non-acidified treatments. However, acidified WSB, CSB, SPB and VWB caused greater increase in mean emergence time from 10.55 to 11.38, 11.24, 11.26 and 11.22 respectively (Fig. 1b).

The effects of acidified BC treatments on coefficient of uniformity of emergence (CUE) of maize seedlings were statistically significant as compared to control and non-acidified treatments. All acidified treatments except RSB caused significant increase in coefficient of uniformity of emergence as compared to control and non-acidified treatments. Acidified WSB, CSB and SPB caused an increase in CUE to 0.14, 0.14, 0.14 respectively (Fig. 1c).

All acidified BCs except CSB and PMB caused a decrease in shoot length of maize seedlings as compared to non-acidified BCs. Acidified WSB and VWB caused a decrease in shoot length to 16.08 and 17.64 cm. However, statistical analysis showed that differences among various non-acidified BCs were not significant as compared to control (Fig. 1d).

### Growth parameters

**Fig. 2:**
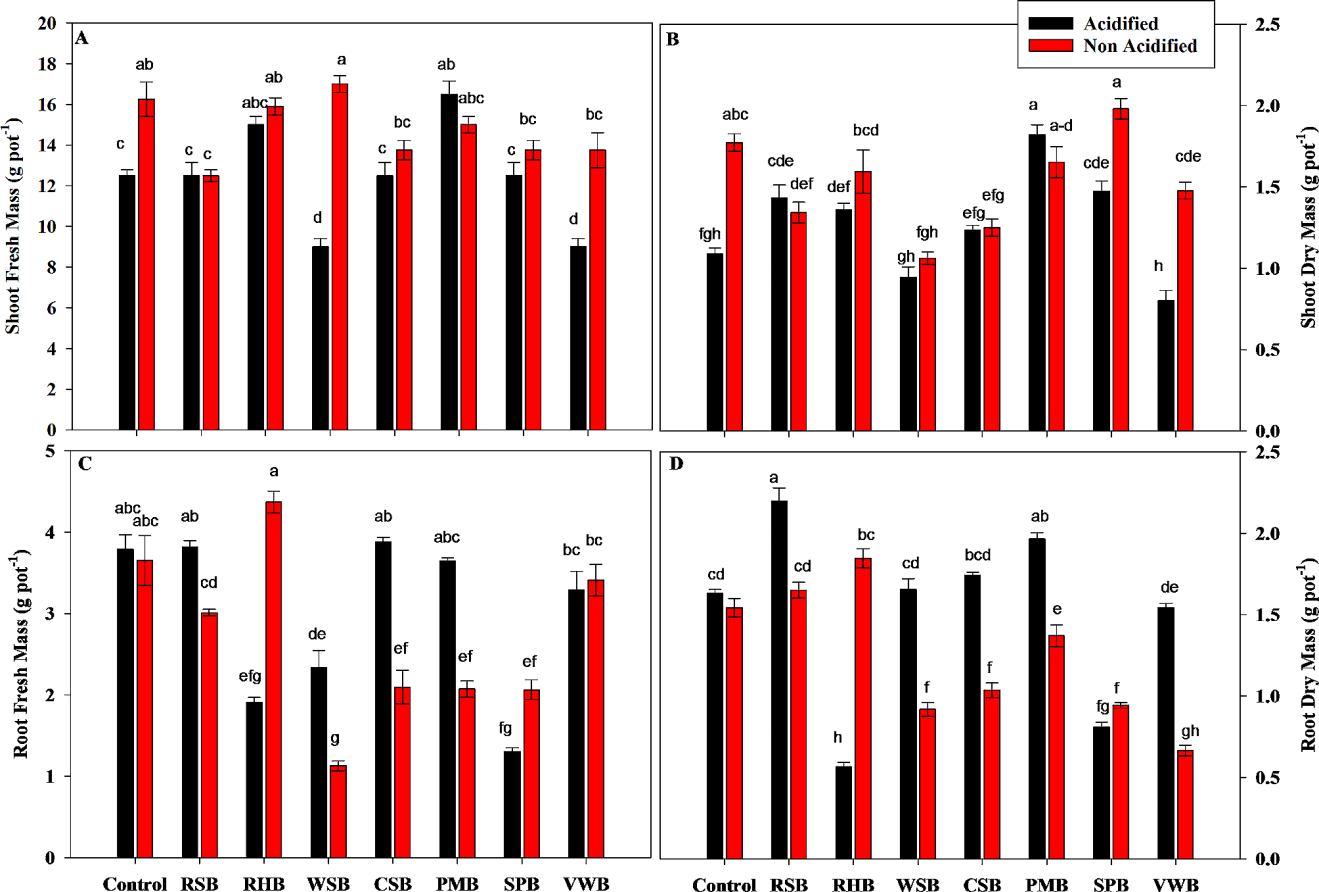
Effects of control, rice straw biochar (RSB), rice husk biochar (RHB), wheat straw biochar (WSB), cotton stalk biochar (CSB), poultry manure biochar (PMB), sugarcane press mud biochar (SPB) and vegetable waste biochar (VWB) on Shoot Fresh Mass **(A)**, Shoot Dry Mass **(B)**, Root Fresh Mass **(C)**, and Root Dry Mass **(D)** of maize plants. Different letters on bars show significant differences among treatments at both levels of acidification (acidified and non-acidified)

Statistical analysis showed that all the acidified BCs except PMB caused a significant decrease in fresh mass of maize seedlings as compared to non-acidified treatments. Acidified WSB and VWB caused a greater decrease in fresh mass to 9 g. Non-acidified WSB caused a greater increase in fresh mass to 17 g (Fig. 2a).

Statistical analysis showed that all acidified treatments except RSB and PMB caused a significant decrease in dry mass of maize seedlings as compared to non-acidified treatments. The greater decrease occurred with acidified WSB and VWB to 0.94 g and 0.80 g respectively. However, non-acidified SPB caused a greater increase in dry mass to 1.98 g (Fig. 2b).

The effect of all acidified treatments except RHB and SPB caused a significant increase in fresh mass of maize roots as compared to non-acidified treatments. RHB and SPB caused a decrease in fresh mass of roots from 3.65 g to 1.90 g and 1.30 g respectively. However, non-acidified RHB caused a greater increase in fresh mass to 4.37 g (Fig. 2c).

Except RHB and SPB all acidified treatments caused significant increase in dry mass of maize roots as compared to non-acidified treatments. A greater increase was caused by acidified RSB to 1.65 g (Fig. 2d).

### Soil properties

**Fig. 3:**
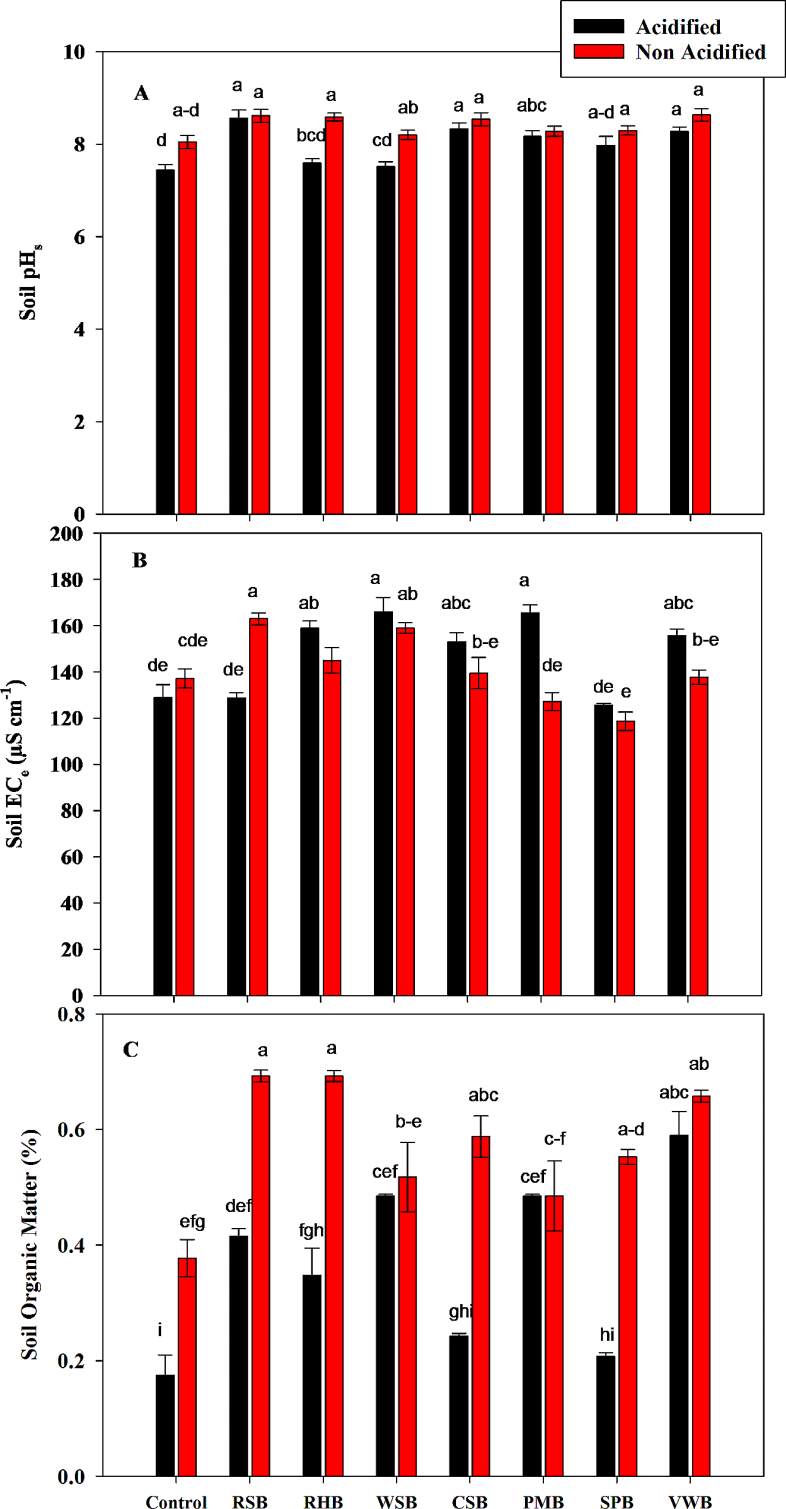
Effects of control, rice straw biochar (RSB), rice husk biochar (RHB), wheat straw biochar (WSB), cotton stalk biochar (CSB), poultry manure biochar (PMB), sugarcane press mud biochar (SPB) and vegetable waste biochar (VWB) on Soil pH **(A)**, Soil Electrical Conductivity **(B)**, and Soil Organic Matter **(C)**. Different letters on bars show significant differences among treatments at both levels of acidification (acidified and non-acidified)

Statistical analysis showed that acid treatments caused a decrease in pH of soil as compared to non-acidified treatments. Non-acidified RSB, RHB, CSB and VWB caused an increase in soil pH from 8.04 to 8.61, 8.59, 8.54 and 8.38, respectively. However, differences in soil pH among various acidified and non-acidified BC treatments were not significant (Fig. 3a).

Statistical analysis showed that all acidified treatments except RSB caused significant increase in soil EC as compared to control and non-acidified treatments. Acidified WSB BC caused greater increase in soil EC to 166 µS/m (Fig. 3b).

Statistical analysis showed that all acidified treatments caused a significant decrease in organic matter contents of soil as compared to non-acidified treatments. Acidified RHB, CSB and SPB caused a greater decrease in organic matter contents to 0.35, 0.24 and 0.21%, respectively. However, all acidified and non-acidified treatments caused significant increase in organic matter content of soil compared to control treatments. Acidified VWB and non-acidified RSB and RHB caused an increase in organic matter contents to 0.59 and 0.69% respectively (Fig. 3c).

**Fig. 4:**
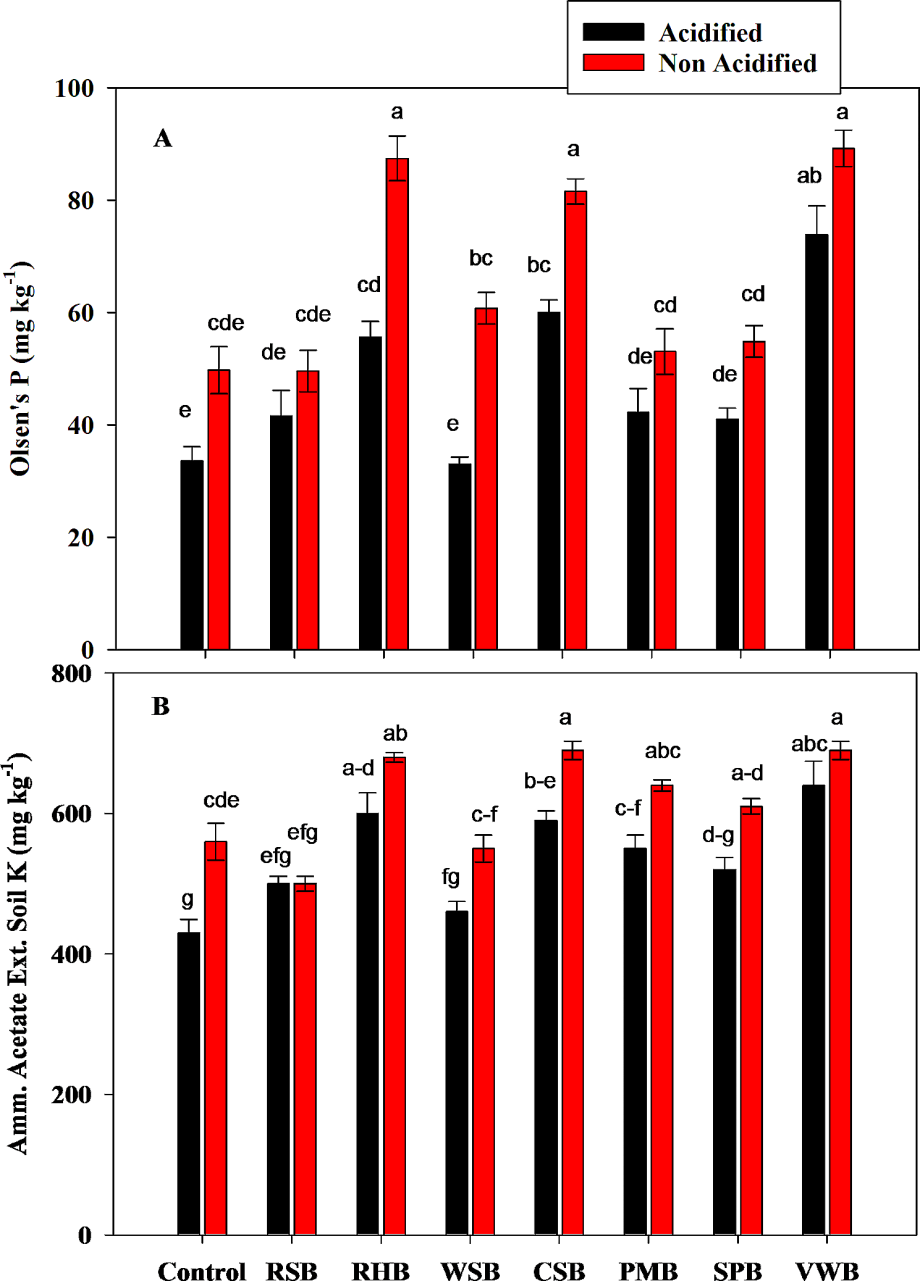
Effects of control, rice straw biochar (RSB), rice husk biochar (RHB), wheat straw biochar (WSB), cotton stalk biochar (CSB), poultry manure biochar (PMB), sugarcane press mud biochar (SPB) and vegetable waste biochar (VWB) on concentration of Soil Olsen’s P **(A)**, and Ammonium Extractable Potassium in Soil **(B)**. Different letters on bars show significant differences among treatments at both levels of acidification (acidified and non-acidified)

There was a significant effect of acidic and non-acidic BC treatments on soil Olsen’s P. All acidified treatments caused a significant decrease in Olsen’s P as compared to non-acidified treatments. However, non-acidified RHB, CSB and VWB caused a greater increase in Olsen’s P from 49.77 to 87.46, 81.58 and 89.27 mg/kg respectively (Fig. 4a). Statistical analysis showed that all acidified treatments except RSB caused a significant decrease in ammonium acetate extractable K as compared to non-acidified treatments. Acidified WSB caused a greater decrease in K concentration to 460 mg/kg However, when compared with control treatments, all BC treatments except non-acidified RHB and WSB caused a significant increase in soil potassium contents (Fig. 4b).

### Elemental concentrations in plant samples

**Fig. 5:**
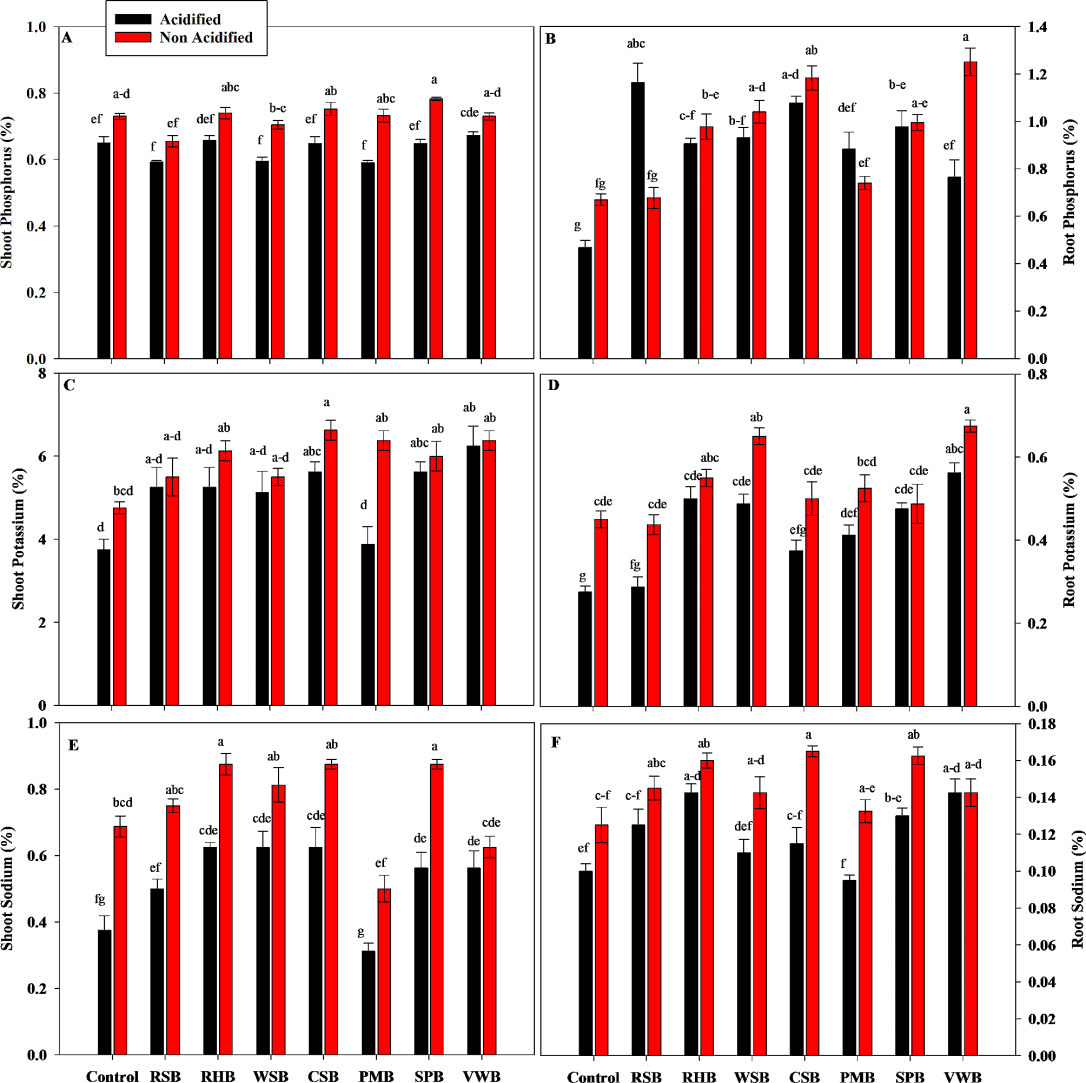
Effects of control, rice straw biochar (RSB), rice husk biochar (RHB), wheat straw biochar (WSB), cotton stalk biochar (CSB), poultry manure biochar (PMB), sugarcane press mud biochar (SPB) and vegetable waste biochar (VWB) on Shoot Phosphorus **(A)**, Root Phosphorus **(B)**, Shoot Potassium **(C)**, Root Potassium **(D)**, Shoot Sodium **(E)**, and Root Sodium **(F)** of maize plants. Different letters on bars show significant differences among treatments at both levels of acidification (acidified and non-acidified)

The effects of acidified BC treatments on P concentration of maize plants was statistically significant. Acidified treatments caused decrease in P as compared to non- acidified treatments. Non-acidified SPB caused a greater increase in P from 0.73 to 0.78% (Fig. 5A). All acidified BCs except RSB and SPB caused significant decrease in P of maize roots as compared to non-acidified BCs. However, non-acidified VWB caused a greater increase in P from 0.67 to 1.25% (Fig. 5B).

Similarly, there was a significant decrease in the K concentration of maize plants in acidified BC treatments as compared to non-acidified BCs. Acidified PMB caused a greater decrease in shoot K concentration to 3.875%. However, differences among various treatments were not statistically significant (Fig. 5C). Statistical analysis showed that all acidified treatments also caused a significant decrease in K in maize roots as compared to non-acidified treatments. Acidified RSB caused a greater decrease in root K to 0.29%. However, all BC treatments caused an increase in K concentration as compared to control treatments. Greater increase was caused by non-acidified VWB to 0.675% (Fig. 5D).

The sodium concentration in maize plants was also significantly affected by the BC treatments. The acidified BCs caused a significant decrease in Na concentration of plants as compared to non-acidified treatments. Non-acidified RHB, CSB and SPB caused greater increase in Na from 0.69 to 0.88% (Fig. 5E). Statistical analysis showed that all acidified BCs except VWB caused significant decrease in Na of maize roots as compared to non-acidified treatments. Acidified PMB caused a greater decrease in Na by 0.09%. However, all BC treatments caused an increase in Na as compared to control treatments (Fig. 5F).

## Discussion

BCs are known to affect plant growth and yield parameters of various crop plants differently, mainly depending on the feedstock type and preparation conditions. In a study, Rogovska et al. [46] reported that BC extracts slightly increased shoot length compared to nutrient-rich solutions. In our study, we used solid BC prepared from various organic feedstock, which have different physicochemical properties than the extracts. We found that none of the BCs negatively affected shoot length and biomass of maize seedlings and roots. However, acidified WSBC and VWBC slightly reduced these parameters. Previous studies showed that BCs may improve soil properties due to porous nature of BCs [47]. All BCs used in our experiment were in alkaline pH shown in Table 1. Previous studies also stated that application of BCs caused increase in soil pH due to presence of negatively charged surfaces which imparts alkaline effects [48]. In our experiment, we used alkaline soil and applied acidified and non-acidified BCs at the rate of 1% (30 g/3kg of soil). Lentz and Ippolito [49] showed that acidic BCs caused slight decrease in pH of calcareous soils when applied at a lower rate. Similar results were found in the case of acidified treatments. Acidified BCs caused slight decrease in pH as compared to non-acidified BCs. Antal and Gronli [50] found that basic cations accumulated in BCs during pyrolysis. Specifically, all our treatments, except non-acidified PMB, SPB and acidified RSB, SPB caused an increase in EC of soil. The decrease in EC of RSB, PMB and SPB may also be due to the loss of mineral contents during pyrolysis.

All acidified and non-acidified BC treatments caused potential positive effects on germination parameters of maize seedlings. This positive effect may be attributed to the high nutrient contents of BCs used in the study (Table 1). Previous studies suggest that BCs produced from variety of organic materials may contain plant available nutrients in varying concentration [6] and may positively influence seed germination of different crops. Ippolito et al. [51] reported that acidic BCs become more beneficial in calcareous soils due to reduction in nutrient losses. They also found that acidic BCs may reduce the losses of nitrate nitrogen through leaching. Previous studies revealed that acidic BCs had capacity to adsorb NH_4_^+^ nitrogen from the soil and supply it to the plants. Therefore, slight increase in germination parameters in some of our acidified treatments as compared to non-acidified treatments may be due to decrease in nutrient losses.

**Fig. 6:**
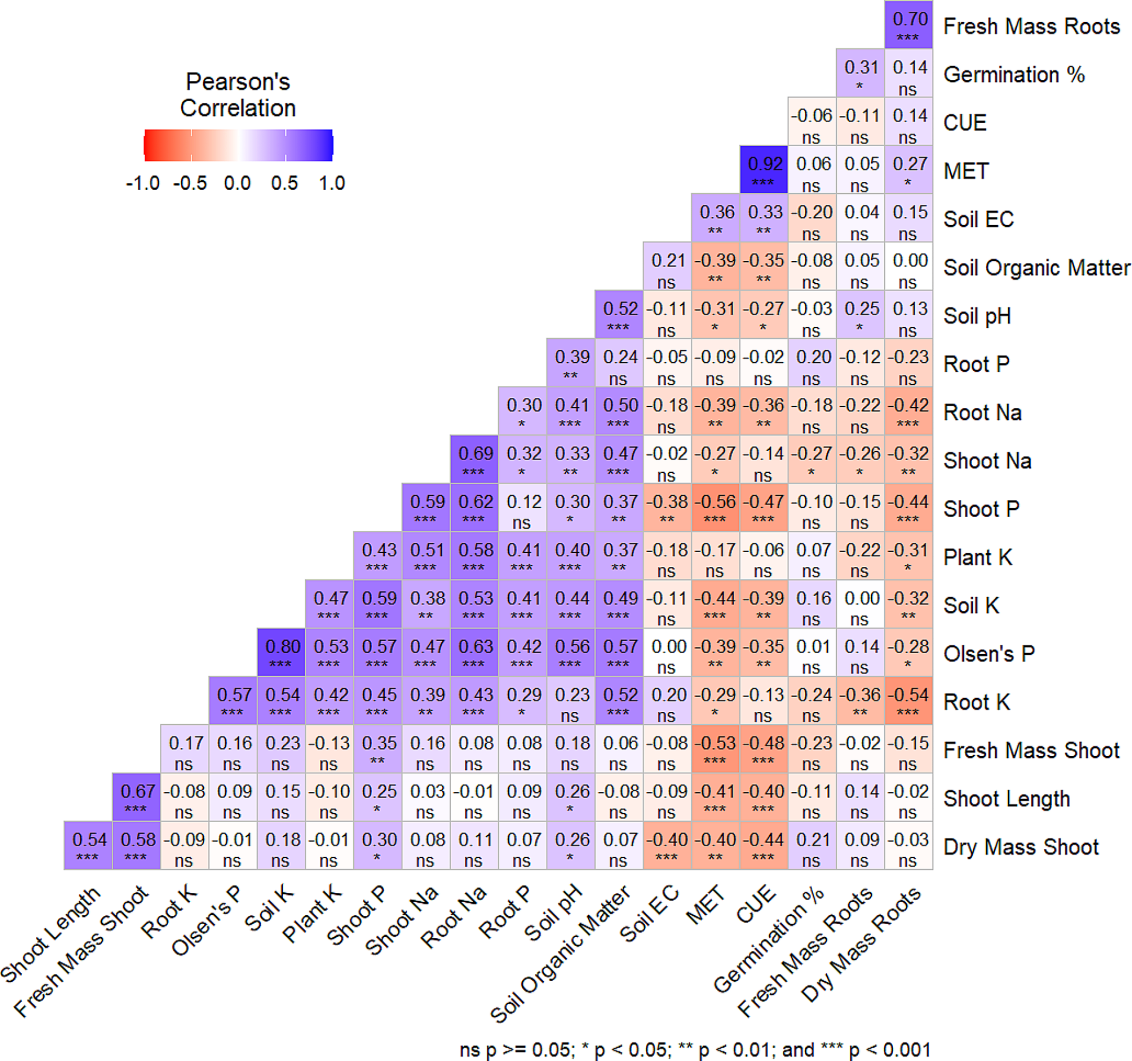
Pearson correlation between the studied parameters in maize growing in different types of biochars. Light Purple shows weak positive correlation, medium purple shows moderate positive correlation, and dark purple indicates strong positive correlation. The different intensity of reddish color shows negative correlation. The white color represents no correlation (value denoted with ns). The asterisks show significance, for example, *p < 0.05, **P < 0.01, and ***p < 0.001)

The Pearson correlation between studied parameters shows significant and positive correlation between soil nutrients and growth parameters (Fig. 6). The soil pH had positive correlation with soil and plant nutrients, while negative correlation with CUE, MET and EC which indicates that change in soil pH due to acidified BCs also caused change in the germination parameters.

In addition to the nutrient contents, BCs may also contain some toxic components which have negative impact on germination of seeds and root growth [21, 52]. Therefore, to assess the presence of either toxic or beneficial compounds in BCs, germination tests must be conducted. Bargmann et al. [53] found higher seed germination in BC treated soils than in un-treated (control) soils. Conversely, Free et al. [54] observed that BCs did not alter the germination and growth of maize seedlings.

Most of the agricultural soils in Pakistan are deficient in organic matter contents (less than 1%); attributed to the high temperature and low rainfall. Such arid environmental condition is unfavourable for general agricultural production. Addition of BCs to soil may intrinsically increase the levels of organic carbon and further help to adsorb organic matter present in the soils [55]. Smebye et al. [46 ] found that acid treatment of BC (0.1 N HCl) caused decrease in the solubility of organic matter contents of soils. Our treatments showed a similar result. Acidified BC treatments had caused a significant decrease in organic matter contents of soil as compared to non-acidified treatments. This was possibly due to enhanced microbial activity due to acidification caused by the acidified BCs. Furthermore, the acidified BCs can enhance the degradation of larger organic compounds into simpler ones that are prone to rapid decomposition [56]. The release of cations from acidified BCs may also interact with organic matter and compete at binding sites, thus influencing the stability SOM [57]. However, when compared with control treatments, all BC treatments caused a significant increase in organic matter contents of soil.

Previously, it was investigated that BCs produced from variety of organic materials may have greater ability to supply essential nutrients present in their ash contents [58]. Among all the essential nutrients, Ca, Mg and K salts are commonly present in the ash contents of BCs. However, carbon contents and plant available nutrient contents of different BCs vary with the type of feedstock used. BCs having alkaline pH tend to accumulate carbon and calcium contents than hydrogen, oxygen, nitrogen, and phosphorus contents [59]. Zhai et al. [60] found that acid modified BCs tend to decrease the P contents and other nutrients of soil than the unmodified BCs. They also stated that acid treatment may remove the ash contents of BCs which mostly constitute 70% of total P and other minerals. In the present experiment, the effects of acidified BCs on Olsen’s P, ammonium extractable K and Na contents of soil, were similar with these findings. However, the non-acidified BCs supplied sufficient nutrients such as P, and K in soil as well as facilitating them for their uptake by maize seedlings.

## Conclusions

The present study investigated the effects of acidified BCs (prepared using multiple sources) on maize seed germination, growth, and soil properties in alkaline-calcareous soil. The study highlighted significant influence of BC types and acid modification on the investigated parameters, addressing knowledge gap in the literature.  Interestingly, acidification significantly affected the characteristics of BCs as compared to the control BCs. The effectiveness of BC_s_ in promoting seed germination, plant biomass, nutrient uptake, and soil properties was influencedby the origin of feedstocks used to make the specific BC. Acidification of BC_s_  delivered better maize growth and biomass accumulation. None of the BCs (acidified and non-acidified) used in present experiment had observable negative effects on alkaline soil conditions and the maize germination processes. However, the acidification of all BCs especially wheat straw BC, cotton stalks biochar and sugarcane press-mud biochar delivered better germination parameters of maize seedlings compared to non-acidified treatments. This study highlighted the strategic application of BCs in cultivation , especially for alkaline soils. Moving forward, more research is needed to understand the long-term effects of modified BCs on nutrient dynamics in different soils. In addition, the possible effects of BC application timings, application rates, particle size, and crop species have to be evaluated systemtically 

## Data Availability

All data supporting the findings of this study are available within the paper.
